# Reinfection with SARS-CoV-2: Discrete SIR (Susceptible, Infected, Recovered) Modeling Using Empirical Infection Data

**DOI:** 10.2196/21168

**Published:** 2020-11-16

**Authors:** Andrew McMahon, Nicole C Robb

**Affiliations:** 1 Department of Physics University of Oxford Oxford United Kingdom; 2 Warwick Medical School University of Warwick Coventry United Kingdom

**Keywords:** infectious disease, SARS-CoV-2, COVID-19, SIR, modeling, reinfection

## Abstract

**Background:**

The novel coronavirus SARS-CoV-2, which causes the COVID-19 disease, has resulted in a global pandemic. Since its emergence in December 2019, the virus has infected millions of people, caused the deaths of hundreds of thousands, and resulted in incalculable social and economic damage. Understanding the infectivity and transmission dynamics of the virus is essential to determine how best to reduce mortality while ensuring minimal social restrictions on the lives of the general population. Anecdotal evidence is available, but detailed studies have not yet revealed whether infection with the virus results in immunity.

**Objective:**

The objective of this study was to use mathematical modeling to investigate the reinfection frequency of COVID-19.

**Methods:**

We have used the SIR (Susceptible, Infected, Recovered) framework and random processing based on empirical SARS-CoV-2 infection and fatality data from different regions to calculate the number of reinfections that would be expected to occur if no immunity to the disease occurred.

**Results:**

Our model predicts that cases of reinfection should have been observed by now if primary SARS-CoV-2 infection did not protect individuals from subsequent exposure in the short term; however, no such cases have been documented.

**Conclusions:**

This work concludes that infection with SARS-CoV-2 provides short-term immunity to reinfection and therefore offers useful insight for serological testing strategies, lockdown easing, and vaccine development.

## Introduction

The novel coronavirus SARS-CoV-2 is thought to have originated in China in late 2019, and has since spread globally, resulting in the COVID-19 pandemic. In the 8 months since the first confirmed case, the virus has resulted in 24 million confirmed infections and over 820,000 deaths, and has caused substantial social and economic damage.

SIR (Susceptible, Infected, Recovered) modeling uses a set of differential equations to determine how the number of infected and recovered individuals changes over time given a specified rate of infection and recovery. It was first used in 1927 by Kermack et al [1] and has since been used to model epidemics from AIDS [2] to SARS (severe acute respiratory syndrome) [3]. Variations of SIR modeling have been used during the COVID-19 pandemic to look at the varying burden on health care systems based on public health intervention [4], the absence of a stable disease-free equilibrium [5], and infection rate [6], as well as the eventual size of the overall pandemic [7]. An extension of the model has also been used to simulate the changing death rate as a function of the number of individuals infected, and it was found that an equilibrium point was reached where there were no further reinfections [8].

In this study, we used an extension to the SIR framework that distinguished between infected and reinfected individuals to model empirical data taken from a compiled COVID-19 data set [9], in order to investigate the reinfection frequency of the disease. We aimed to determine if cases classified as “reinfections” will occur, although to date there is no definitive cases of reinfection reported in the scientific literature.

## Methods

### Data Sources

We used national infection and mortality data from a variety of sources to investigate the reinfection dynamics of SARS-CoV-2. Unless specified, national data on infections and deaths from SARS-CoV-2 were acquired from the Our World in Data database compiled by the Oxford Martin School at the University of Oxford [9]; the hospitalization cases in Switzerland were obtained from the Federal Office of Public Health in Switzerland [10]; the data for the city of New York were obtained from the New York City Health website [11]; the population of New York City was obtained from the 2019 New York census [12]; and the recovery data for Germany was sourced from Trading Economics [13], which obtains its data from the World Health Organization (WHO). For each geographical region, the data were taken from the date of the first recorded infection up until May 17, 2020, when the data were accessed.

### Choice of Geographical Regions

The simulations were initially completed for the United Kingdom, where, at the time the data were accessed, there was a high number of confirmed cases. Australia was selected as an example of a region with low numbers of recorded cases, in order to investigate the limit of expected reinfections; Germany was selected as it was one of the few countries with recorded recovery data; Italy was studied since the number of infections and deaths had peaked by May 17, 2020; Singapore was unique as a city-state so population density for the nation was very high; Switzerland was selected since hospitalization data were available at the time the data were accessed; and the United States as a whole was compared with New York City, which was the worst affected part of the United States at the time.

### Assumptions

A number of assumptions have been made. Where possible, they have been made so that the number of reinfections is underestimated. These assumptions are as follows:

1. There is a large lag time for recovery to take place (28 days) [14,15].
2. The incubation period was modeled as 6 days [16].
3. The model does not consider social distancing or shielding and so assigns an equal probability of an infection to all individuals.
4. Not all infections have been recorded due to lack of testing, misdiagnosis, or asymptomatic infection [17].
5. Infections and recoveries are not necessarily recorded on the date that they first occurred.
6. There is no emigration out of, or immigration into, a population of interest.
7. The model assumes a homogeneous population density, with no societal structure (eg, equal number of residents per household).

### The Model

**Figure 1:**
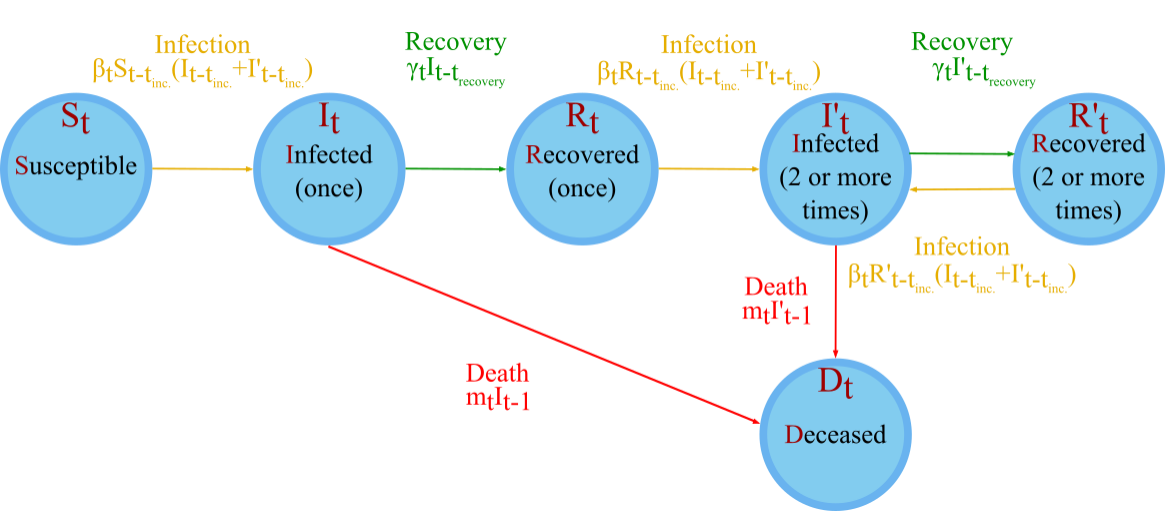
A simple representation of the model. S_t_ represents the number of persons susceptible to infection who have had no prior infections on day t; I_t_ is the number of people currently infected for the first time on day t; R_t_ is the number of people who have recovered once on day t; I’_t_ is the number of people who have been infected 2 or more times and are infected on day t; R’_t_ is the number of people who have recovered 2 or more times and are not infected on day t of the model, and D_t_ is the number of deceased persons on day t of the model. Further symbols are defined in Table 1.

We based our model on the compartmental SIR framework, but differentiated between initial and subsequent infections, resulting in a 6-state model (susceptible, infected, recovered, infected [2 or more times], recovered [2 or more times], and deceased) (Figure 1 and Table 1). The number of infections and deaths each day was taken from national statistics (as described above). Where available, recovery data were used; otherwise, recoveries were modeled with a 28-day lag time (with the number of recoveries representing those individuals who did not die during the 28-day recovery time). “Recovered” individuals were selected stochastically from the populations of the states 28 days prior [14,15], “infected” individuals from the populations of the states 6 days prior (due to the incubation period [16]), and “deceased” individuals from the populations of the states 1 day prior.

**Table 1:** Definition of parameters in the model.

Parameter	Definition
x_i_	Random number
t	The number of days into the simulation; t=1 for the day of the first infection
t_recovery_	The average number of days for recovery (t_recovery_=28)
t_incubation_ = t_inc._	The average number of days before an infection is seen (t_incubation_=6)
t_max_	The number of days over which the simulation is run
S_t_	The number of susceptible individuals on day t
I_t_	The number of infected (once) individuals on day t
R_t_	The number of recovered (once) individuals on day t
I’­_t_	The number of infected (2 or more times) individuals on day t
R’_t_	The number of recovered (2 or more times) individuals on day t
D_t_	The number of deceased individuals from SARS-CoV-2 on day t
Β_t_	The infection rate on day t
γ	The recovery rate on day t
m_t_	The death rate on day t
N	The total population in the model; N = S_t_ + I_t_ + R_t_ + I’_t_ + R’_t_ + D_t_ ∀ t
N_t_^infected^	The number of infected individuals on day t; N_t_^infected^ = I_t_ + I’_t_
N_t_^uninfected^	The number of uninfected individuals on day t; N_t_^uninfected^ = S_t_ + R_t_ + R’_t_
n_t_^infected^	The number of new infections on day t; n_t_^infected^ = β_t_S_t–t_incubation__(I_t–t_incubation__ + I’_t–t_incubation__) + β_t_R_t–t_incubation__(I_t–t_incubation__ + I’_t–t_incubation__) + β_t_R’_t–t_incubation__(I_t–t_incubation__ + I’_t–t_incubation__)
n_t_^recovered^	The number of recovering individuals on day t; n_t_^recovered^ = γ_t_I_t–t_recovery__ + γ_t_I’_t–t_recovery__
n_t_^deaths^	The number of deaths on day t; n_t_^deaths^ = m_t_I_t–1_ + m_t_I’_t–1_
i_t_^first time^	The number of first-time infections on day t (ie, the number of S_t_ → I_t_ transitions on day t)

When using the model, the rates of infection, recovery, and fatality for each state were assumed to be independent of how many infections a host had previously had. The number of susceptible persons at the beginning of the simulation, N, was taken to be the population of the region of interest [9,12]. After all infections, recoveries, and deaths for a day, the number of days into the simulation was increased by one, t → t + 1 up to t_max_. The simulation was repeated 10,000 times to produce expectation values and standard deviations for the number of individuals classified as reinfections.

By pooling the number of cases in the infected (2 or more times), recovered (2 or more times), and deceased (after 2 or more infections) states at the end of the simulation, we calculated an estimate of the number of reinfections that would be expected to occur. This number represents the total population that had passed through the infected (2 or more times) state by the end of the simulation.

Unless otherwise stated, the average recovery time used in the simulations was set as 28 days, as this is greater than the median recovery time suggested in the report of the WHO-China Joint Mission on Coronavirus Disease 2019 [14].

## Results

### Simulations of UK Infection Data Suggest a Small Number of Reinfections Should Have Occurred

**Figure 2:**
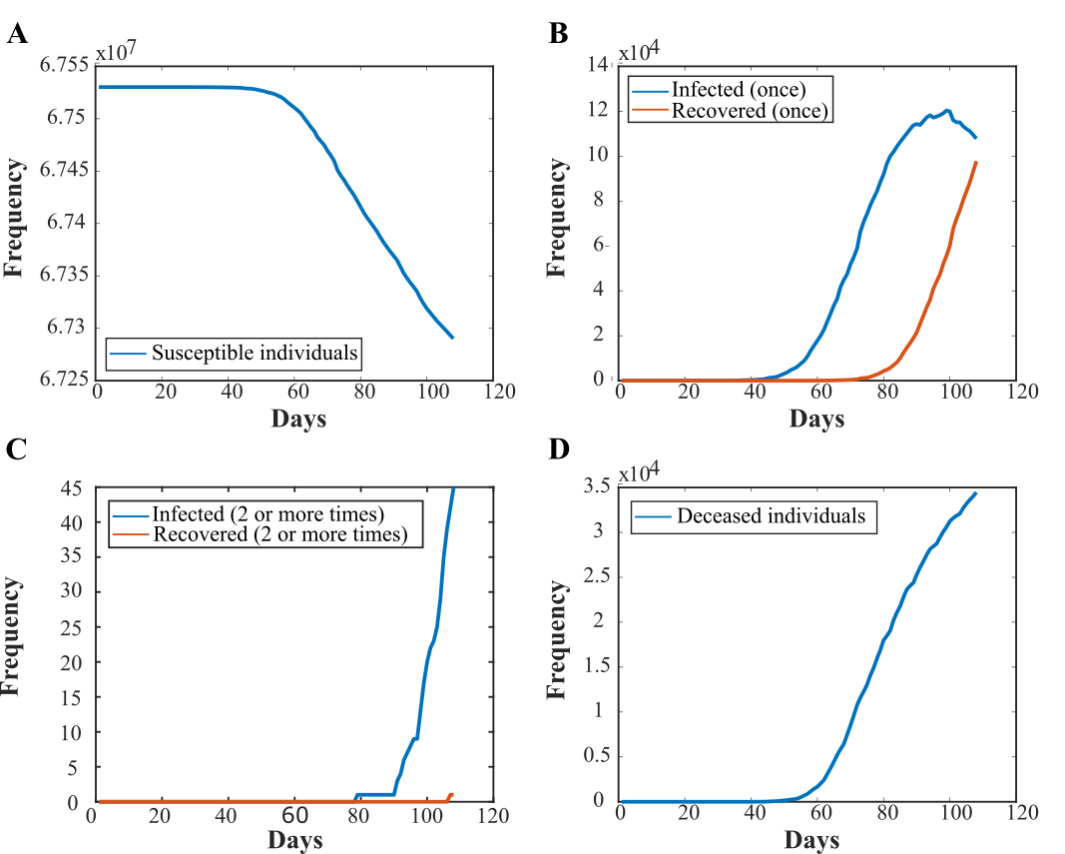
Plots of the populations of each state in the model over the course of a typical simulation, using infection data from the United Kingdom. (A) An example plot of the susceptible population in the model over the course of 106 days (from the first recorded case on February 1 until May 17, 2020, when the data were accessed). (B) An example plot of the populations that are infected for the first time or recovered from a single infection. (C) An example plot of the populations of the simulation that have been reinfected and have recovered from an infection twice. (D) An example plot of the number of deceased individuals through the course of the simulation.

We initially ran the simulation for data in the United Kingdom over the course of 106 days (from the first recorded case on February 1 until May 17, 2020, when the data were accessed). Figure 2 shows how the population of each state in the model changed over the course of a typical simulation. The number of susceptible individuals initially remained steady, until day 55, when there was a sharp decline due to the increase in primary infections (Figure 2A). The number of individuals infected just once started to increase steadily after day 40 and continued to do so throughout the simulation until day 92. After the 28-day lag time, the individuals infected once started to recover, resulting in an increase in the recovered (once) state through to the end of the simulation (Figure 2B). As the number of recovered individuals started to increase, so did the number of people infected for a second time. The number of people recovered for the second time started to increase after the 28-day recovery lag time (Figure 2C). The number of deaths started to rise from day 55 onwards, and fatalities continued to increase through to the end of the simulation (Figure 2D). In the United Kingdom, the number of expected reinfections was calculated to be 43 (SD 7), which makes up 0.018% of the total infections (Table 2). The first reinfection for the United Kingdom was on day 82 (SD 5), corresponding to April 22, 2020.

**Table 2:** The number of predicted reinfections and their standard deviation in different locations worldwide as predicted from the model. Unless otherwise stated, these figures represent simulations using the total number of infections for each region and are modeled without the data on the number of recoveries.

Region	Reinfections, mean (SD)	Infections, N	Reinfections as a % of the total infections
Australia	0.1 (0.3)	7036	0.0014
Germany	20 (5)	174,355	0.011
Germany (with recovery data)	79 (9)	174,355	0.05
Italy	63 (8)	224,760	0.028
New York City	209 (15)	189,027	0.11
New York City (hospitalizations)	7 (3)	48,462	0.004
Singapore	4 (2)	27,356	0.014
Switzerland	4 (2)	30,587	0.013
United Kingdom	43 (7)	240,161	0.018
United States	402 (20)	1,467,884	0.027

### Simulations of Infection Data in Other Regions Show a Similar Trend

The simulations were repeated with data from Australia, Italy, New York City, Singapore, Switzerland, and the United States. The mean number of expected reinfections in each region or country for the 10,000 simulations that were run are shown in Table 2. In all cases, with the exception of Australia, our model predicts that reinfection cases should occur.

### Comparison of Infection and Hospitalization Data in New York City

**Figure 3:**
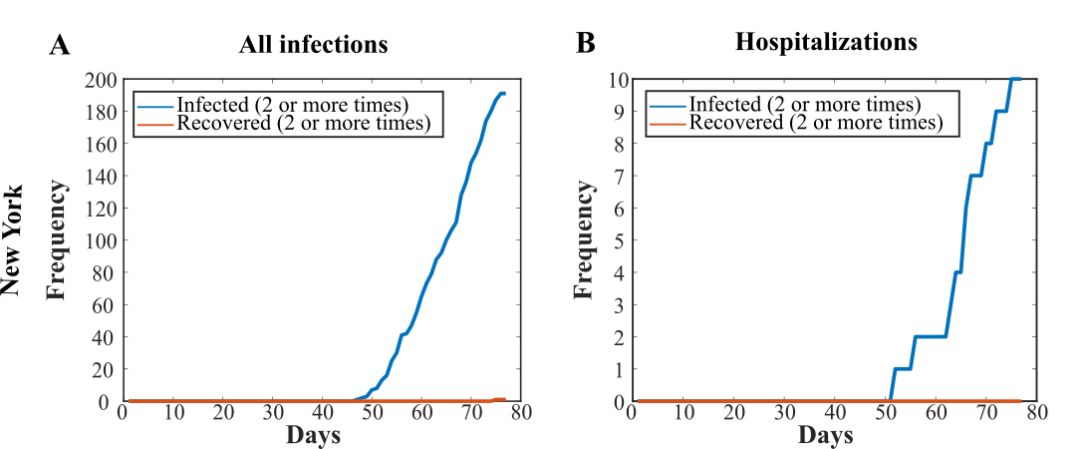
Comparison of total infections versus hospitalization data in New York City. Plots of the infected (2 or more times) and recovered (2 or more times) states for (A) New York using all infection data and (B) New York using only the hospitalization data.

Next, we repeated our simulation for New York City, with the total number of infections replaced by the number of hospitalizations. When we ran the simulation with an input of the total number of infections, the number of secondary infections continued to increase throughout the simulation, when the numbers appear to start to peak (Figure 3A). This was followed by an increase in the number of secondary recoveries after the 28-day recovery lag time. In comparison, the hospitalization data for New York showed no secondary recoveries as the reinfections occurred later into the simulation (Figure 3B). The total number of predicted reinfections from the New York hospitalized data was 12 (SD 4) (Table 2).

### Inclusion of Recovery Data Suggests That Predicted Reinfections Are Underestimated

**Figure 4:**
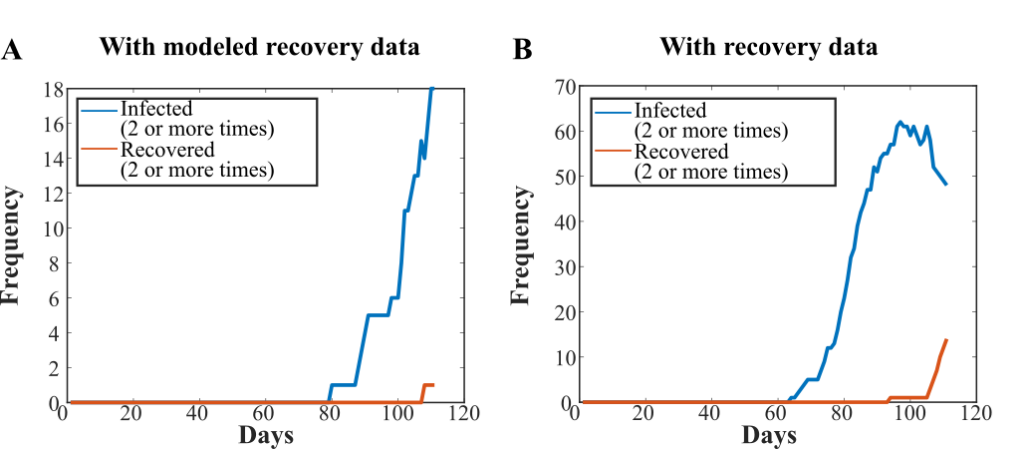
Plots of infected (2 or more times) and recovered (2 or more times) states with (A) modeled recovery data and (B) actual recovery data. Use of actual recovery data from Germany suggests that the number of recovered individuals, and hence reinfections, are underestimated in our model.

Recovery data was sparse or unavailable for most regions, likely due to lack of follow-up testing. Recovery data were available from Germany, and we therefore compared the results of our simulation for Germany with and without the recovery data as an input. The models used a 28-day lag before the recoveries started, meaning very few secondary recoveries took place (Figure 4A and B). There were 73 more reinfections with the reinfection data than with the modeled data (Table 2).

The 28-day lag time used for the modeled recovery data ensured that we underestimated the recovery rate, and therefore the rate of reinfection as well. To investigate a more life-like recovery rate, the United Kingdom simulations were repeated again using the modeled recovery data, while shortening the lag time for recovery. As expected, we found that the rate of reinfection increased as the lag time was decreased from 28 days through to 7 days, as there was a larger population that recovered from a primary infection. With a 7-day lag time, the number of people in the infected (2 or more times) state peaked at day 101 of the simulation (Multimedia Appendix 1). The total number of people reinfected throughout the simulation increased as the lag time decreased, with 43 (SD 7), 83 (SD 9), 139 (SD 12), and 209 (SD 14) reinfections for 28-day, 21-day, 14-day, and 7-day recovery lag times, respectively.

## Discussion

In this work, we have presented a modeling strategy used to determine whether SARS-CoV-2 reinfections can occur. We modeled actual infection and fatality data from different regions around the world and found that all regions investigated, with the exception of Australia, should have recorded cases of reinfections if primary infection with SARS-CoV-2 did not provide some level of immunity. The actual number of cases of reinfection that have been reported in any of these regions or countries to date is zero, suggesting that worldwide, primary SARS-CoV-2 infection provides short-term immunity.

In Australia, the number of confirmed SARS-CoV-2 infections at the time the data were accessed was relatively low [9], possibly due to early social distancing measures, the closing of international borders, and mass testing and tracing measures. The number of modeled reinfections (0.1 [SD 0.3]; Table 2) reflects this, and so even without immunity from infection no reinfections would be expected to occur. Similarly, in Switzerland and Singapore, very low numbers of reinfections were predicted by the model (6.2 [SD 2.5] and 6 [SD 2], respectively; Table 2). It is possible that these very low numbers of reinfection cases could have been missed due to misdiagnosis or lack of follow-up testing. We therefore applied our model to data from Germany [9], Italy [9], New York City [11,12], and the United States as a whole [9], which have recorded far higher numbers of SARS-CoV-2 infections (174,355; 224,760; 189,027; and 1,467,884, respectively, when the data were accessed). The number of reinfection cases predicted for these countries was 30 (SD 6), 89 (SD 9), 335 (SD 18), and 635 (SD 25) for Germany, Italy, New York, and the United States, respectively (Table 2). We conclude that it is therefore very unlikely that all of these predicted cases, if true, were missed due to misdiagnosis or lack of testing.

We also found that rehospitalization cases should have been seen amongst hospitalized cases in New York City—it is unlikely that these cases would be missed as people are processed and tested on admission into hospital. To date, however, no reinfections have verifiably been recorded anywhere in the world. A report from South Korea suggested that 116 patients recovered from COVID-19 had tested positive by RT-PCR (reverse transcription–polymerase chain reaction) for the virus again [18]; however, this has since been explained as the “false-positive” detection of remnants of viral RNA (ribonucleic acid) rather than reactivation or reinfection. The lack of documented reinfections suggests that short-term immunity to the virus is produced by an initial infection, although our model cannot predict whether this immunity will last over longer timescales.

Our results are supported by a number of animal challenge studies, which also show that immunity to SARS-CoV-2 can be conferred. A study in rhesus macaques showed that, following initial viral clearance, the monkeys showed a reduction in their median viral load in comparison with primary infection when rechallenged with SARS-CoV-2 [19]. Similarly, Ryan et al [20] demonstrated that rechallenged ferrets were fully protected from acute lung pathology. An adenovirus-vector vaccine tested on rhesus macaques elicited a humoral and cellular response that, on challenge with the virus, proved to significantly reduce the viral load in bronchoalveolar lavage fluid and respiratory tract tissue [21]. However, a longitudinal study by Seow et al [22] showed that the immunity conferred against SARS-CoV-2 may only be short term. Our model proposes that reinfection cases should have already started to appear by April 2020, suggesting a possible lower limit for immunity duration.

A report from the WHO-China Joint Mission on Coronavirus Disease 2019 estimated the recovery time for SARS-CoV-2 infection to be 2 weeks for mild cases and 3-6 weeks for severe or critical cases [14]; based on this we used a long (28 days) recovery lag time in the modeled data. Comparison with real-world recovery data from Germany suggested that the actual recovery time may be significantly shorter, giving rise to an underestimation of the reinfection rate in our modeled data. This was supported by an increase in the number of predicted reinfections in the UK simulations when we used a shorter recovery lag time of 7, 14, or 21 days. In addition, there were no allowances in our model for transmission being localized to regions smaller than a nation or city; the daily infection data were likely to be only a fraction of the total number of infections due to asymptomatic or mild infections not being recorded, and infections were recorded on the date of testing, not the actual date of infection. We also note that significant differences in testing, reporting, and shielding of the vulnerable exist between the different regions in this study and that a large number of COVID-19 cases were missed in every region of interest (eg, in Geneva, unreported cases were estimated to be 11.6 infections per reported infection from April 6 to May 9 [17]). In every region, we expect that the impact on our simulation would be to underestimate the number of reinfections. Taken together, this suggests that the actual reinfection rate would be significantly higher than that predicted by our model if there was no immunity conferred by prior infection.

Our model has a number of limitations, including the lack of modeling of any social structure, the fact that individuals who have been infected may change their shielding behaviors, differing recovery times from person to person, and missing information regarding immigration into and out of regions of interest. In spite of this, the results documented here provide strong evidence, based on real data, to suggest that that there is at least short-term immunity conferred by an initial infection of SARS-CoV-2. This has implications for serological testing strategies, lockdown easing timescales, and vaccine development. Our modeling strategy can also be extended to understand the reinfection dynamics of future pandemics.

## Appendix

Multimedia Appendix 1 Plots of infected (2 or more times) and recovered (2 or more times) populations in the United Kingdom when the lag time used was (A) 28 days, (B) 21 days, (C) 14 days, and (D) 7 days. Use of lower recovery lag times leads to an increase in the number of expected reinfections.

## Abbreviations

RNA: ribonucleic acid
RT-PCR: reverse transcription–polymerase chain reaction
SARS: severe acute respiratory syndrome
SIR: Susceptible, Infected, Recovered
WHO: World Health Organization

## References

[1] Kermack WO, McKendrick AG, Walker GT (1997). A contribution to the mathematical theory of epidemics. Proc R Soc Lond A.

[2] Victor Okhuese Alexander (2020). Estimation of the Probability of Reinfection With COVID-19 by the Susceptible-Exposed-Infectious-Removed-Undetectable-Susceptible Model. JMIR Public Health Surveill.

[3] Ng TW, Turinici G, Danchin A (2003). A double epidemic model for the SARS propagation. BMC Infect Dis.

[4] Ming W, Zhang C Breaking down of healthcare system: mathematical modelling for controlling the novel coronavirus (2019-nCoV) outbreak in Wuhan, China. bioRxiv.

[5] Victor A (2020). Mathematical predictions for COVID-19 as a global pandemic. SSRN Journal.

[6] Nesteruk I Statistics based predictions of coronavirus 2019-nCoV spreading in mainland China. medRxiv.

[7] Batista M Estimation of the final size of the COVID-19 epidemic. medRxiv.

[8] Okhuese Alexander Victor (2020). Estimation of the Probability of Reinfection With COVID-19 by the Susceptible-Exposed-Infectious-Removed-Undetectable-Susceptible Model. JMIR Public Health Surveill.

[9] Ritchie H, Roser M, Ortiz-Ospina E, Hasell J (2020). Coronavirus pandemic (COVID-19) - Country by country. Our World in Data.

[10] (2020). New coronavirus: Situation in Switzerland. Federal Office of Public Health.

[11] (2020). Covid-19: Data. NYC Health.

[12] United States Census Bureau (2019). QuickFacts: Richmond County (Staten Island Borough), New York; New York County (Manhattan Borough), New York; Bronx County (Bronx Borough), New York; Kings County (Brooklyn Borough), New York; Queens County (Queens Borough), New York. Census.gov.

[13] (2020). Germany Coronavirus Recovered. Trading Economics.

[14] (2020). Report of the WHO-China Joint Mission on Coronavirus Disease 2019 (COVID-19). World Health Organization.

[15] Bi Q, Wu Y, Mei S, Ye C, Zou X, Zhang Z, Liu X, Wei L, Truelove SA, Zhang T, Gao W, Cheng C, Tang X, Wu X, Wu Y, Sun B, Huang S, Sun Y, Zhang J, Ma T, Lessler J, Feng T (2020). Epidemiology and transmission of COVID-19 in 391 cases and 1286 of their close contacts in Shenzhen, China: a retrospective cohort study. The Lancet Infectious Diseases.

[16] Backer JA, Klinkenberg D, Wallinga J (2020). Incubation period of 2019 novel coronavirus (2019-nCoV) infections among travellers from Wuhan, China, 20-28 January 2020. Euro Surveill.

[17] Stringhini S, Wisniak A, Piumatti G, Azman AS, Lauer SA, Baysson H, De Ridder D, Petrovic D, Schrempft S, Marcus K, Yerly S, Arm Vernez I, Keiser O, Hurst S, Posfay-Barbe KM, Trono D, Pittet D, Gétaz L, Chappuis F, Eckerle I, Vuilleumier N, Meyer B, Flahault A, Kaiser L, Guessous I (2020). Seroprevalence of anti-SARS-CoV-2 IgG antibodies in Geneva, Switzerland (SEROCoV-POP): a population-based study. The Lancet.

[18] Cha S, Smith J (2020). Explainer: South Korean findings suggest 'reinfected' coronavirus cases are false positives. Reuters.

[19] Chandrashekar A, Liu J, Martinot Amanda J, McMahan Katherine, Mercado Noe B, Peter Lauren, Tostanoski Lisa H, Yu Jingyou, Maliga Zoltan, Nekorchuk Michael, Busman-Sahay Kathleen, Terry Margaret, Wrijil Linda M, Ducat Sarah, Martinez David R, Atyeo Caroline, Fischinger Stephanie, Burke John S, Slein Matthew D, Pessaint Laurent, Van Ry Alex, Greenhouse Jack, Taylor Tammy, Blade Kelvin, Cook Anthony, Finneyfrock Brad, Brown Renita, Teow Elyse, Velasco Jason, Zahn Roland, Wegmann Frank, Abbink Peter, Bondzie Esther A, Dagotto Gabriel, Gebre Makda S, He Xuan, Jacob-Dolan Catherine, Kordana Nicole, Li Zhenfeng, Lifton Michelle A, Mahrokhian Shant H, Maxfield Lori F, Nityanandam Ramya, Nkolola Joseph P, Schmidt Aaron G, Miller Andrew D, Baric Ralph S, Alter Galit, Sorger Peter K, Estes Jacob D, Andersen Hanne, Lewis Mark G, Barouch Dan H (2020). SARS-CoV-2 infection protects against rechallenge in rhesus macaques. Science.

[20] Ryan K, Bewley K, Fotheringham S, Brown P, Hall Y, Marriott AC, Tree JA Dose-dependent response to infection with SARS-CoV-2 in the ferret modelvidence of protection to re-challenge. bioRxiv.

[21] van Doremalen N, Lambe T, Spencer A, Belij-Rammerstorfer S, Purushotham JN, Port JR, Avanzato V ChAdOx1 nCoV-19 vaccination prevents SARS-CoV-2 pneumonia in rhesus macaques. bioRxiv.

[22] Seow J, Graham C, Merrick B, Acors S, Steel KJA, Hemmings O, O'Bryne A Longitudinal evaluation and decline of antibody responses in SARS-CoV-2 infection. medRxiv.

