# Supplementary material for: Reinfection with SARS-CoV-2: Discrete SIR (Susceptible, Infected, Recovered) Modeling Using Empirical Infection Data
Source: JMIR Public Health Surveill. 2020 Nov 16;6(4):e21168. doi: 10.2196/21168 (PMC7674142; doi:10.2196/21168)
Supplement: Multimedia Appendix 1 [file publichealth_v6i4e21168_app1.pdf]

Supplementary Material

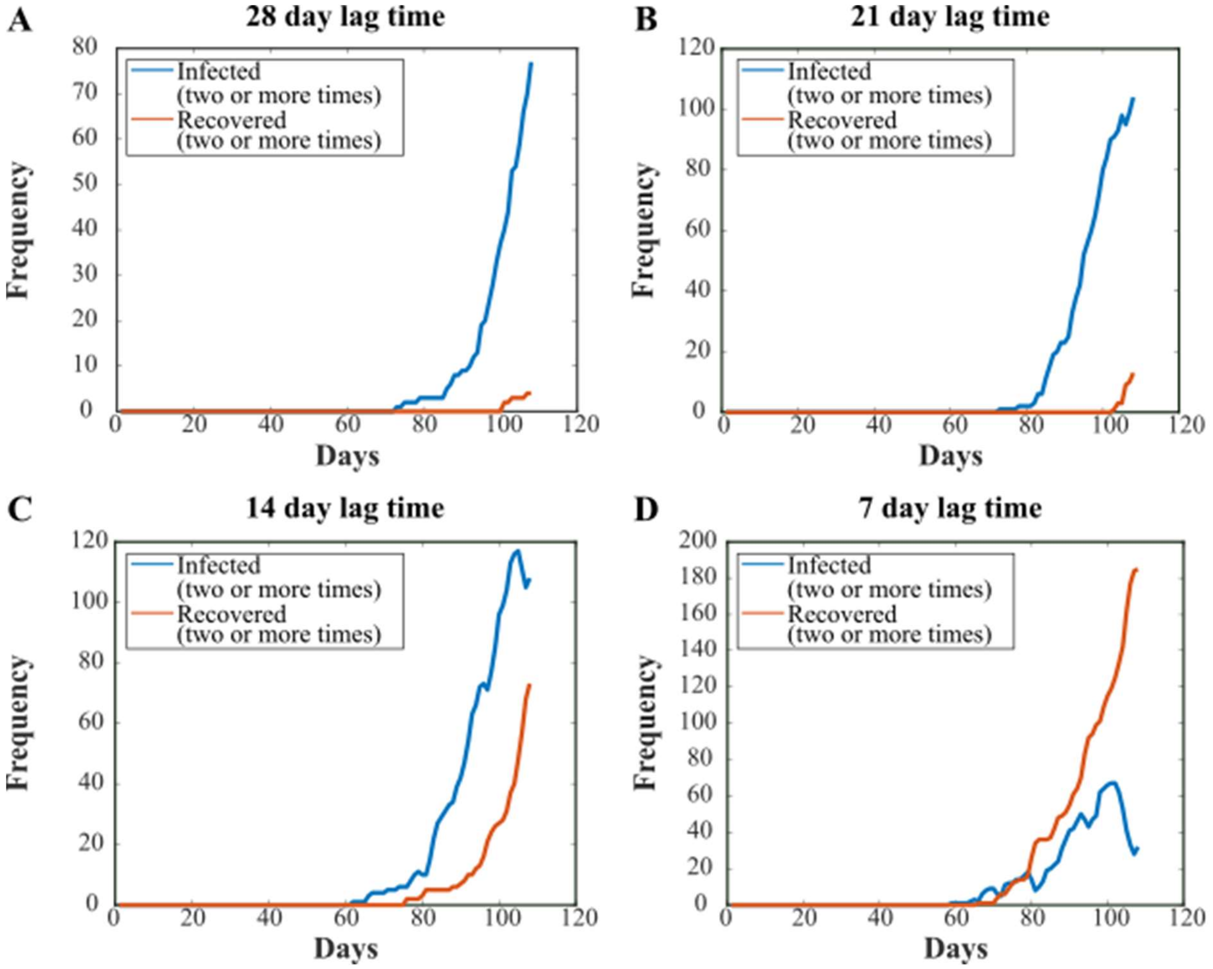

Supplementary Figure 1. Use of lower recovery lag times leads to an increase in the number of expected reinfections. Plots of Infected (two or more times) and Recovered (two or more times) populations in the United Kingdom when the lag time used was A) 28 days B) 21 days C) 14 days and D) 7 days.
